# Modulation of Myocardial Mitochondrial Mechanisms during Severe Polymicrobial Sepsis in the Rat

**DOI:** 10.1371/journal.pone.0021285

**Published:** 2011-06-21

**Authors:** Mani Chopra, Honey B. Golden, Srinivas Mullapudi, William Dowhan, David E. Dostal, Avadhesh C. Sharma

**Affiliations:** 1 Department of Biomedical Sciences, Texas A&M Health Science Center Baylor College of Dentistry, Dallas, Texas, United States of America; 2 Department of Molecular Cardiology, Cardiovascular Research Institute, College of Medicine, Texas A&M Health Science Center, Temple, Texas, United States of America; 3 Department of Biochemistry and Molecular Biology, The University of Texas Medical School at Houston, Houston, Texas, United States of America; 4 Cardionome Laboratory, Department of Pharmaceutical Sciences, School of Pharmacy, Philadelphia College of Osteopathic Medicine, Suwanee, Georgia, United States of America; Rutgers University, United States of America

## Abstract

**Background:**

We tested the hypothesis that 5-Hydroxydecanoic acid (5HD), a putative mitoK_ATP_ channel blocker, will reverse sepsis-induced cardiodynamic and adult rat ventricular myocyte (ARVM) contractile dysfunction, restore mitochondrial membrane permeability alterations and improve survival.

**Methodology/Principal Findings:**

Male Sprague-Dawley rats (350–400 g) were made septic using 400 mg/kg cecal inoculum, ip. Sham animals received 5% dextrose water, ip. The Voltage Dependent Anion Channels (VDAC1), Bax and cytochrome C levels were determined in isolated single ARVMs obtained from sham and septic rat heart. Mitochondria and cytosolic fractions were isolated from ARVMs treated with norepinephrine (NE, 10 µmoles) in the presence/absence of 5HD (100 µmoles). A continuous infusion of 5HD using an Alzet pump reversed sepsis-induced mortality when administered at the time of induction of sepsis (−40%) and at 6 hr post-sepsis (−20%). Electrocardiography revealed that 5HD reversed sepsis-induced decrease in the average ejection fraction, Simpsons+m Mode (53.5±2.5 in sepsis and 69.2±1.2 at 24 hr in sepsis+5HD vs. 79.9±1.5 basal group) and cardiac output (63.3±1.2 mL/min sepsis and 79.3±3.9 mL/min at 24 hr in sepsis+5HD vs. 85.8±1.5 mL/min basal group). The treatment of ARVMs with 5HD also reversed sepsis-induced depressed contractility in both the vehicle and NE-treated groups. Sepsis produced a significant downregulation of VDAC1, and upregulation of Bax levels, along with mitochondrial membrane potential collapse in ARVMs. Pretreatment of septic ARVMs with 5HD blocked a NE-induced decrease in the VDAC1 and release of cytochrome C.

**Conclusion:**

The data suggest that Bax activation is an upstream event that may precede the opening of the mitoK_ATP_ channels in sepsis. We concluded that mitoK_ATP_ channel inhibition via decreased mitochondrial membrane potential and reduced release of cytochrome C provided protection against sepsis-induced ARVM and myocardial contractile dysfunction.

## Introduction

Despite advances in critical care medicine research, death due to sepsis and associated pathologies has increased by alarming proportions in the last two decades. It is well recognized that severe sepsis is associated with cardiac failure and high mortality rates ranging from 30–60% [1]. Alterations in sepsis, septic shock and related pathologies involving mitochondrial ultrastructural changes and oxidative mechanisms have received major attention in the last few years. In a model of endotoxemia, Crouser et al. demonstrated that endotoxin-induced mitochondrial damage was related to an imbalance in mitochondrial respiration [2]. The severity of sepsis has been shown to correlate with mitochondrial damage and bioenergetic dysfunction in both human and experimental models [2], [3], [4], [5]. In another model of bacterial challenge, the oxidation of myocardial mitochondrial protein and lipid was observed at 4 and 24 hr, suggesting outer mitochondria membrane (OMM) damage [6], [7].

For several years, our laboratory has produced evidence of molecular apoptotic mechanisms in sepsis-induced myocardial and ARVM dysfunction [8], [9]. Our data also demonstrated the role of mitochondrial-mediated intrinsic apoptosis cascade and stress-mediated mitogen-activated protein kinases in the regulation of sepsis-induced adult rat ventricular myocyte (ARVM) dysfunction [9], [10], [11], [12]. In experimental endotoxemia, mitochondrial dysfunction has been characterized by mitochondrial membrane potential collapse and transitional changes in mitochondrial membrane permeability, along with the release of cytochrome C [8], [13], [14], [15]. Earlier, we reported that a progressive decline in myocardial performance at 3 and 7 days in a hyperdynamic model of sepsis is associated with increased levels of proapototic caspase-3, increased B-cell leukemia (Bcl_2_)-associated protein×(Bax)/Bcl_2_ ratio and release of cytochrome C [8]. Mitochondrial outer membrane permeabilization (MOMP) is controlled by the translocation of Bax on OMM [16]. Disturbance of OMM leads to MOMP and release of a large number of intramitochondrial proteins, including cytochrome C, via the formation of permeabilization pores primarily composed of Voltage Dependent Anion Channels (VDACs) [17]. Besides VDACs, including VDAC1, which are present on OMM, the inner mitochondrial membrane (IMM) bilayer also possess mitochondrial K_ATP_ (mitoK_ATP_) channels [18]. The Kir subunits of mitoK_ATP_ channels are closely associated with sulphonylurea protein SUR2A, which is a regulatory protein for the passage of pharmacological agents through these channels [19]. Several researchers have shown that mitoK_ATP_ channels are directly activated by diazoxide and blocked by 5-hydroxydecanoate (5HD) [18]. Even though both diazoxide and 5HD have been shown to be only partially specific to the mitoK_ATP_ channels [20], 5HD apparently remains the most selective antagonist of the mitoK_ATP_ channels available.

NE is a positive inotrope used in the current therapy of sepsis to maintain hemodynamic support for the ICU patients but has been shown to produce cardiodynamic dysfunction in septic animals [21]. Our previous findings have demonstrated that NE produces blunted contractile response to ARVMs along with up regulation of mitochondrial-driven apoptotic cascade [22]. In the present study we speculated that OMM damage during sepsis could be accompanied by the opening of mitoK_ATP_ channels that play a critical role in the transport of ATP across mitochondrial membranes. We hypothesized that 5HD, a putative mitoK_ATP_ channel blocker, will ameliorate sepsis-induced cardiodynamic and ARVM contractile dysfunction, restore mitochondrial membrane permeability alterations and improve the survival rate. Therefore, we determined the effect of mitoK_ATP_ inhibition on sepsis-induced mortality, myocardial and ARVM contractile dysfunction by using 5HD. The effect of 5HD was examined in presence and absence of NE on ARVM contractility and release of cytochrome C, and levels of Bax and VDAC1. In addition, we determined whether modulation of the mitoK_ATP_ channels affected the IMM events leading to the release of cytochrome C in the myocardium in a severe septic rat model.

## Results

### Effect of 5HD on sepsis-induced hypotension, hypothermia and mortality

Unlike the sham rats, the septic animals displayed all the behavioral signs of sepsis including piloerection, periocular discharge, severe diarrhea and lethargy. Upon postmortem analysis, the peritoneal cavity revealed the presence of ascites, which are pus-filled lesions on various organs (stomach wall, liver and kidneys) indicative of peritoneal infection (personal observations; Chopra and Sharma, 2007).

**Figure 1:**
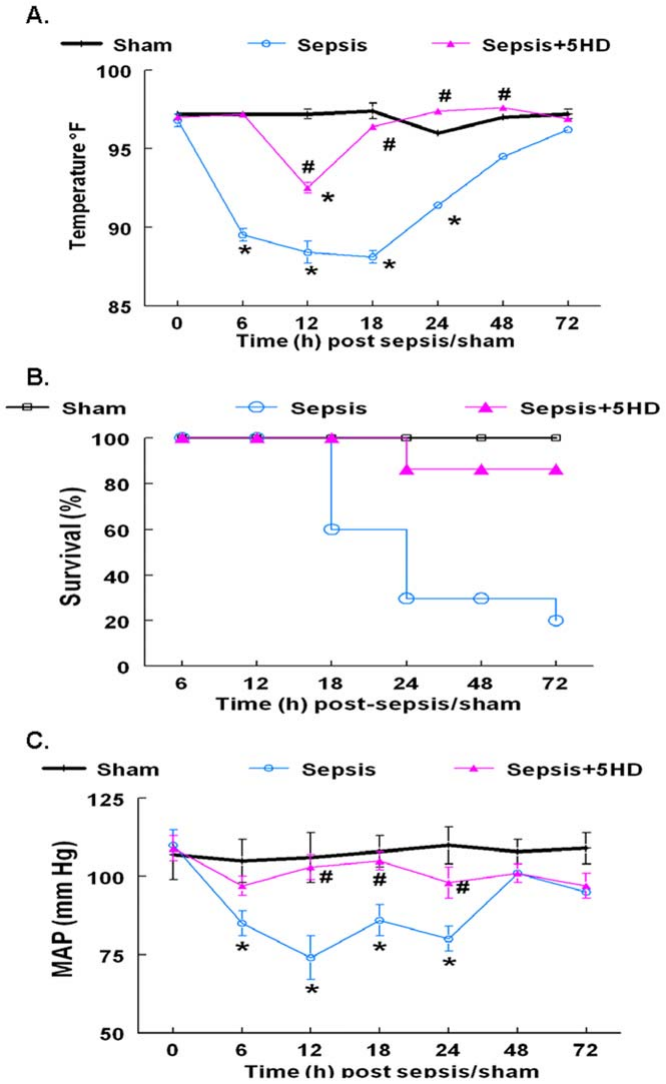
Effect of 5HD, administered at the time of sham/sepsis induction, on hemodynamics and survival in the rat. The effect of 5HD on the, (**A**) rectal temperature, (**B**) survival and (**C**) Mean Arterial Pressure, MAP of the septic rats. Sham (N = 10), sepsis (N = 10); sepsis+5HD (N = 7). The values in the sepsis group at 48 and 72 hr include only two surviving animals.*P≤0.05 compared to the sham group and # P≤0.05 compared to the sepsis group at respective time points.

The septic animals exhibited significant hypothermia (Fig. 1A) and hypotension (Fig. 1C) during 6–12 hr post-sepsis compared to the sham group. The septic animals displayed 30% survival (70% mortality) up to 24 hr post-sepsis (Fig. 1B). The 5HD induced increase in survival was found statistically significant compared to the sepsis group (P<0.05). The 5HD treatment at the time of the induction of sepsis dramatically increased the survival of the septic animals (Fig. 1A) and improved the overall animal movement in the cage. Importantly, 5HD treatment also reversed the sepsis-induced hypothermia (Fig. 1B) and hypotension (Fig. 1C) within 6–24 hr.

**Figure 2:**
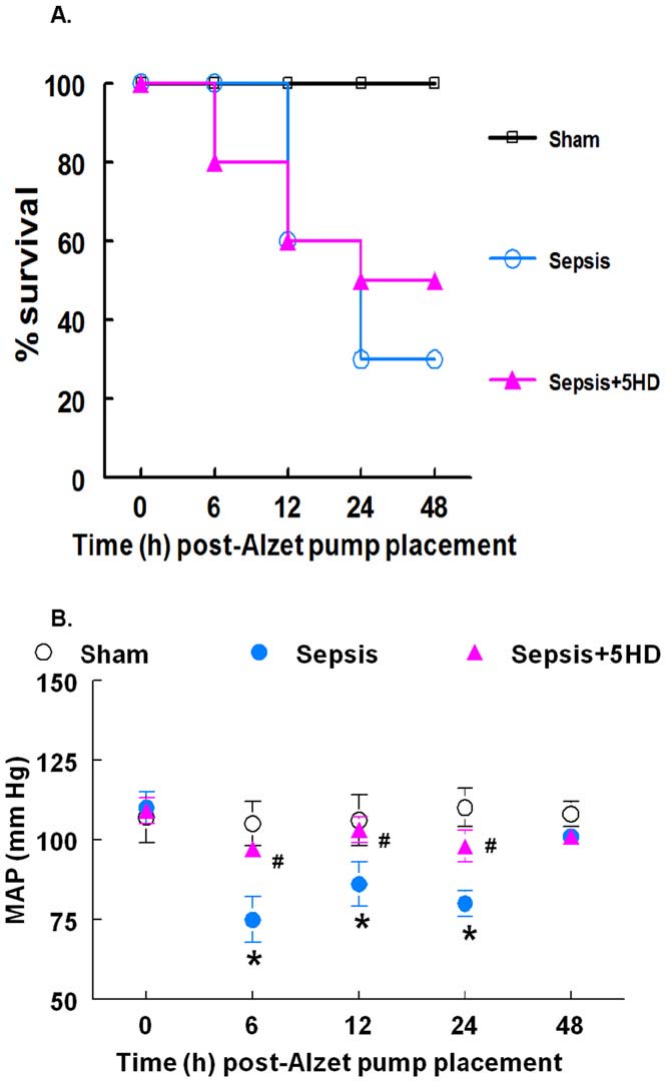
Effect of 5HD, administered at 6 hr post-sham/sepsis induction, on hemodynamics and survival in the rat. The effect of 5HD on (**A**) % survival at 48 hr and (**B**) MAP at 0, 6, 12, 24 and 48 hr post-Alzet pump (containing 5HD or saline) placement. *P≤0.05 compared to the 0 hr values and # P≤0.05 compared to the sepsis group at respective time points (N = 10 in each group).

The percent mortality in the 5HD treated (at 6 hr post-sepsis) septic animals was 50% compared to 70% mortality (30% survival) in the sepsis group (untreated animals) at 24 hr post-sepsis (Fig. 2A). The septic animals treated with saline at 6-hr post-sepsis underwent a significant decrease in the mean arterial pressure (MAP) at 6, 12 and 24 hr post-Alzet pump placement compared to the sham group (Fig. 2B). The MAP in the septic animals that survived >24 h post sepsis was found to be similar to that in the sham group (however, only the data from two animals in the sepsis group were available at this time point). The 5HD treatment at 6 hr post sepsis produced a significant increase in MAP at 6-, 12- and 24-hr post-Alzet pump placement compared to the saline-treated sepsis group (Fig. 2B).

### Effect of 5HD on cardiodynamics and the concentration of TNF-α

**Figure 3:**
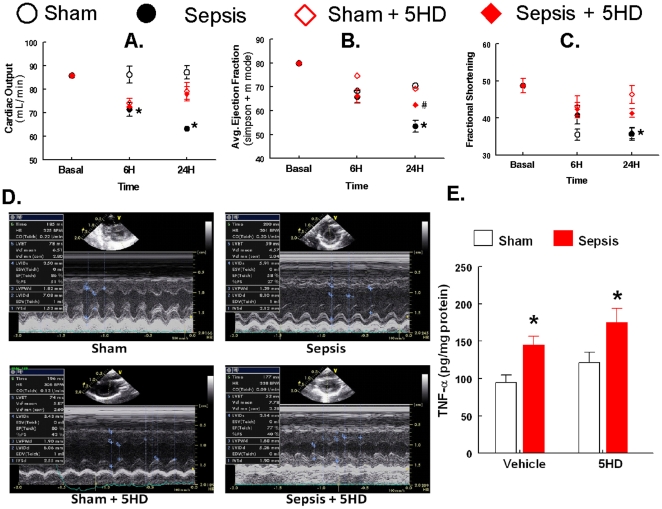
Effect of 5HD, administered at the time of sham/sepsis induction, on cardiodynamics and TNF-α levels in the rat. The effect of 5HD (100 µg/100 µL) on the cardiac output, CO (**A**); average ejection fraction, EF (**B**); fractional shortening, FS (**C**) measured using Vivid I at baseline (basal), 6 and 24 hr post-treatment by Alzet pump (**D**); the representative m-mode echocardiography recorded to calculate Simpson's ejection fraction in the rat. (**E**) the effect of 5HD on the concentration of TNF-α in the myocardial supernatant obtained from the heart collected at 24 hr post-sepsis in the rat. *P≤0.05 compared to the basal values and # P≤0.05 compared to the sepsis group at respective time points. (N = 6 in each group).

Cardiodynamic parameters such as cardiac output (CO), ejection fraction (EF) and fractional shortening (FS) were measured to determine the *in vivo* effect of 5HD treatment in the septic rats (Fig. 3). The saline-infused septic animals exhibited a significant reduction in CO (71±3 ml/min, p≤0.05) compared to the basal levels (93±4 ml/min, p≤0.05) at 6 hr. This reduction continued for 24 hr (63±1.32 ml/min, p≤0.05) compared to the basal levels (93±4 ml/min, p≤0.05) (Fig. 3A). The 5HD treatment of the septic animals (at the time of induction of sepsis) significantly increased the previously decreased CO (p≤0.05) in the animals at 24 hr (Fig. 3A). The ejection fraction, a measure of the efficacy of the heart, was found to be significantly decreased (53±4%, p≤0.05) compared to the basal levels (80±2%, p≤0.05) in the septic rats at 24 hr (Fig. 3B). Interestingly, the 5HD treatment of the septic animals dramatically increased the EF (62±2%, p≤0.05) compared to its respective sepsis group (53±4%, p≤0.05), an indicator of improved cardiac performance (Fig. 3B). The representative m-mode echocardiography for the calculation of Simpson's ejection fraction in the rat was performed, which corroborated and supported the above-mentioned EF results (Fig. 3D). Fractional shortening, an index of left ventricle (LV) contractility, was found to be significantly decreased at 24 hr (p≤0.05) in the septic rats compared to the basal levels (49±2%, p≤0.05) (Fig. 3C). No significant difference was observed at any time point in any group.

To determine whether 5HD treatment affects the local inflammatory response in the septic rat heart, we determined the concentration of TNF-α in the supernatant of the harvested heart samples in the sham and sepsis groups (Fig. 3E). This result suggests that the upregulation of TNF-α is an early event in the extrinsic apoptotic cascade and is independent of the alterations due to 5HD treatment in the septic ARVMs.

**Figure 4:**
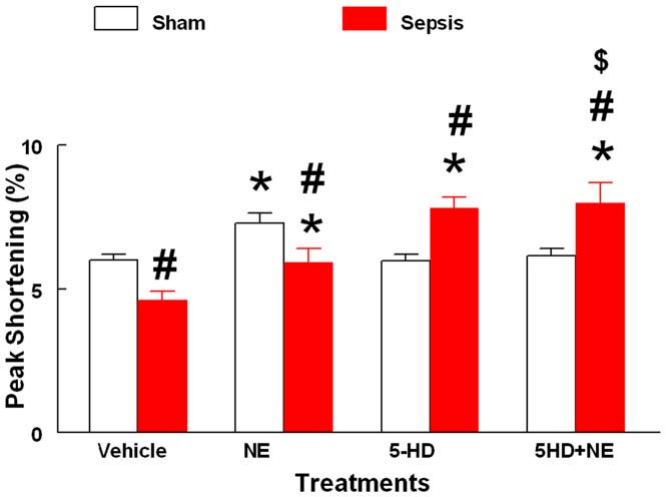
Effect of 5HD on ARVM contractility. The effect of 5HD on percent peak shortening in sham and septic ARVMs at 12 hr post-incubation in the culture medium (N = at least 50 ARVMs isolated from 5 rat hearts in each treatment group). The data are expressed as mean ± SEM. *p≤0.05 compared to the respective vehicle treatment group; and # p≤0.05 compared to the respective vehicle treatment group; $ p≤0.05 compared to the respective NE-treated sham/sepsis groups.

### Effect of 5HD on sepsis-induced depressed contractility in ARVMs

Sepsis produced a significant decrease in the percentage peak shortening (% PS) of ARVMs compared to the sham group (Fig. 4). In addition, NE produced a significant (P<0.05) increase in the % PS in the sham and septic ARVMs compared to their respective vehicle treatment groups. However, the effect of NE in the septic ARVMs was significantly lower compared to the sham NE group (P<0.05, Fig. 4). Interestingly, the 5HD-treated septic ARVMs produced a positive inotropic response shown by a significant increase in the % PS compared to its vehicle and sham-treated groups. Further, the addition of NE in the 5HD-treated septic ARVMs accentuated the % PS response significantly (P<0.05) compared to its respective vehicle, sham and NE-treated groups alone (Fig. 4).

**Figure 5:**
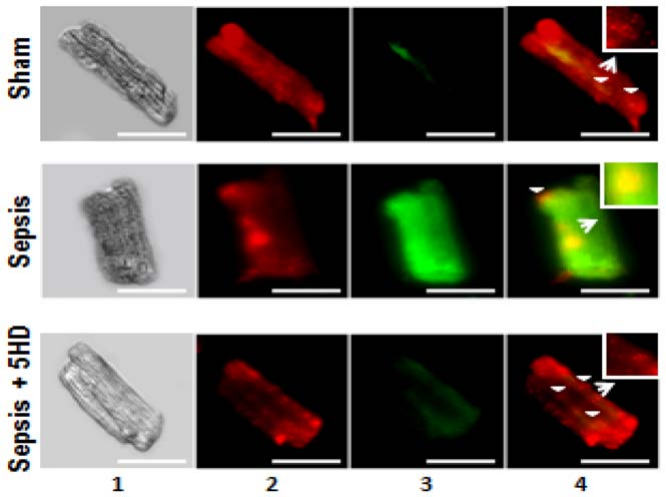
Effect of 5HD on mitochondrial membrane potential (ΔΨm) in ARVMs. Mitochondrial ΔΨm was examined in sham and septic ARVMs and visualized using a fluorescent microscope. Representative photomicrographs (magnification 40×; scale bar, 75 µm) of sham and septic ARVMs treated in the presence and absence of 5HD are stained with JC-1 reagent. Lane 1 exhibits the ARVMs studied under a light microscope. The red (JC-1 aggregates in the mitochondria; lane 2) and green (JC-1 monomers in cytoplasm; lane 3) fluorescence was recorded and merged using an image software (lane 4). The white arrowheads depict the location of mitochondria in the ARVMs (which were zoomed 10 times and shown in the boxedsquare).

### Effect of 5HD on sepsis-induced mitochondrial ΔΨm collapse in ARVMs

A fluorescent cationic dye, JC-1, was employed to determine the alterations in the mitochondrial membrane ΔΨm in the ARVMs (Fig. 5). The sham ARVMs exhibited red fluorescence as the JC-1 dye accumulated in the mitochondria, indicative of the healthy or normal cells (upper panel; Fig. 5). However, the septic ARVMs exhibited green fluorescence as the JC-1 dye remained in the monomeric form, typical of mitochondrial membrane potential collapse and an early sign of apoptosis (middle panel; Fig. 5). However, the addition of 5HD in the septic ARVMs reduced the green fluorescence and exhibited more red fluorescent aggregates in the mitochondria, suggesting a restorative effort of 5HD on the mitochondrial membrane ΔΨm (lower panel; Fig. 5).

### Effect of 5HD, *in vivo*, on VDAC1 and sepsis-induced increased release of Cytochrome C in ARVMs

**Figure 6:**
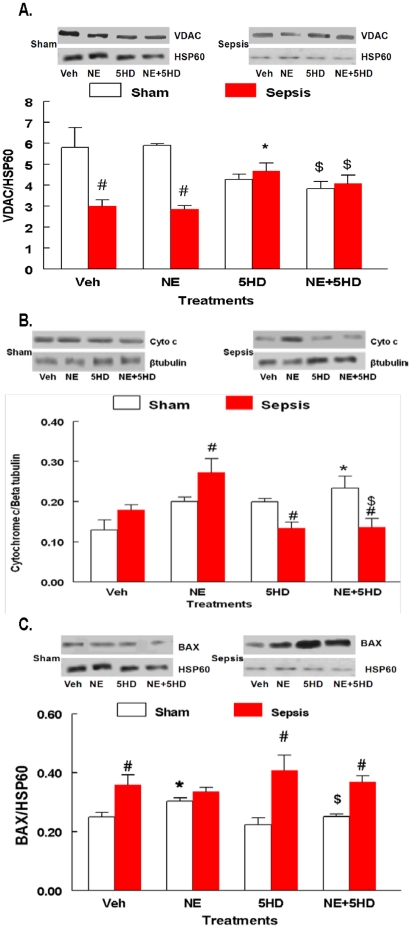
Effect of 5HD on VDAC1, release of cytochrome C and Bax. The effect of 5HD on the expression of mitochondrial VDAC1 (**A**), cytosolic Cytochome C (**B**) and Bax (**C**) in the sham and septic ARVMs (N = 4 in each treatment group). *P<0.05 compared to the respective vehicle-treated sham and sepsis groups; #P<0.05 compared to the respective sham treatment groups. $P<0.05 compared to the respective sham/sepsis NE group.

The mitochondrial fractions obtained from the vehicle and NE-treated septic ARVMs produced a significant decrease in the VDAC 1 protein levels compared to its respective sham treatment groups (Fig. 6A). However, mitochondrial fractions from the 5HD-treated septic ARVMs exhibited a significant increase in the VDAC 1 protein levels. Further, the addition of NE to the 5HD-treated groups produced a significant increase in the VDAC 1 protein levels compared to the respective NE- and vehicle treated sham and sepsis groups, suggesting that 5HD blocked the sepsis-induced downregulation of mitochondrial VDAC 1. The presence of mitochondrial fractions and equal loading of the samples was confirmed by HSP60 (Fig. 6A).

The NE-treated septic ARVMs produced a significant increase in the cytochrome C protein levels compared to the sham treatment group (Fig. 6B). However, 5HD treatment in the presence and absence of NE in the septic ARVMs resulted in a significant reduction in the cytochrome C levels compared to the vehicle-treated group, suggesting that inner mitochondrial membrane integrity was maintained. The cytoplasmic fractions, as well as equal loading of the samples, were confirmed by β-tubulin.

The mitochondrial fractions obtained from septic ARVMs produced a significant increase in the Bax protein levels compared to the respective sham-treated group (Fig. 6C). NE-treatment significantly increased Bax proteins in the sham ARVMs compared to the vehicle–treated sham group but was not different than NE-treated sepsis group. Treatment of ARVMs with 5HD in sepsis groups produced a significant increase in Bax compared to the respective sham group. Treatment with 5HD+NE displayed a significant increase in the Bax levels in sepsis group compared to the respective vehicle-treated sham group (Fig. 6C). These data indicate that Bax regulation during sepsis could be an event prior to the release of cytochrome C from the mitochondria.

### Sepsis downregulates colocalization of ANT with VDAC1 in isolated purified mitochondrial preparation (PMPs) obtained from septic ARVMs

The co-immunoprecipitation technique was performed to determine the interaction between VDAC 1, ANT and SUR 2 during sepsis.

**Figure 7:**
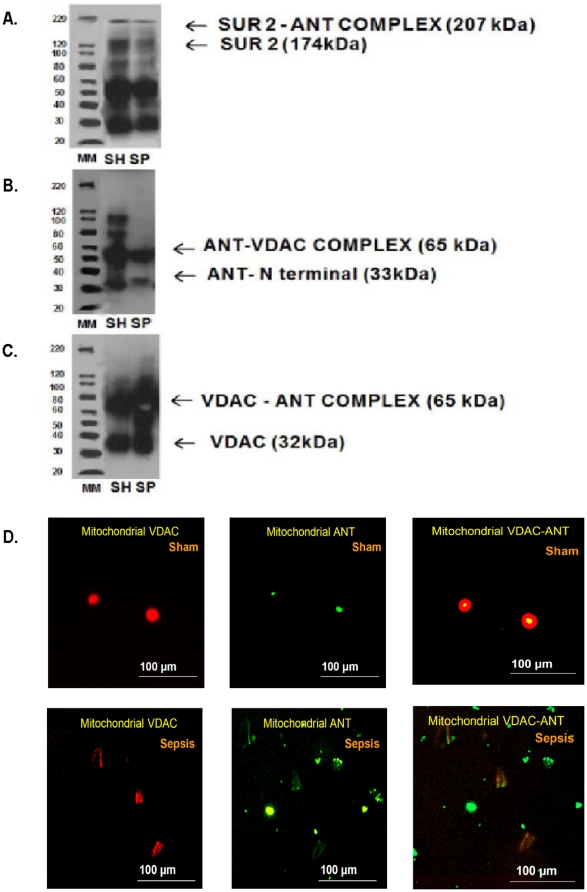
Binding of ANT with SUR/VDAC1. Co-immunoprecipitation assay was performed to determine binding of ANT with SUR 2 (A), VDAC 1 (VDAC) (B), and VDAC1 with ANT (C) using the Dynabeads Protein G method (Invitrogen). The experiments were conducted in the PMPs obtained from the sham and sepsis ARVMs (N = 3 in each treatment group). **D**. Colocalization of ANT with VDAC1 was performed using a fluorescent microscope in the purified mitochondrial fraction obtained from the sham and septic ARVMs. Representative photomicrographs (Magnification, 10×; Scale bar = 100 µm) were treated with anti-rabbit VDAC1 and anti-goat ANT antibodies to observe their co-localization in the mitochondrial fractions. The co-localized expression of VDAC1 (555 nm, red fluorescence, left panel), and ANT (488 nm, green fluorescence, middle panel) was observed in yellowish-orange fluorescence (right panel).

As shown in Fig. 7A, cellular protein, SUR2 was co-immunoprecipitated with ANT protein in both sham and septic ARVMs. By gel electrophoretic analysis, the top band corresponding ∼207 kDa protein was identified as SUR2-ANT complex. The bottom band corresponding ∼174 kDa was identified as SUR2. We confirmed that SUR 2 protein level was elevated in sham PMPs compared to sepsis group. Similarly, in Figs. 7B and 7C; ANT protein expression, a bottom band corresponding ∼33 kDa and VDAC1 protein expression, a bottom band corresponding ∼32 kDa were elevated in sham PMPs compared to septic group. Interestingly, it was observed that VDAC1-ANT complex expression was faint in the septic PMPs compared to the sham group (Fig. 7C).

The purified mitochondrial preparations (PMPs) obtained from sham ARVMs exhibited a brilliant yellow fluorescence (colocalization) of ANT and VDAC1 under a fluorescent microscope (Fig. 7D). However, the PMPs obtained from septic ARVMs exhibited dispersed VDAC 1 (red fluorescence, 555 nm) and ANT (green fluorescence, 488 nm), suggesting that sepsis hinders the formation of VDAC 1 and ANT complex in the mitochondria (Fig. 7D).

### Effect of 5HD on sepsis-induced mitochondrial cristae deformation

**Figure 8:**
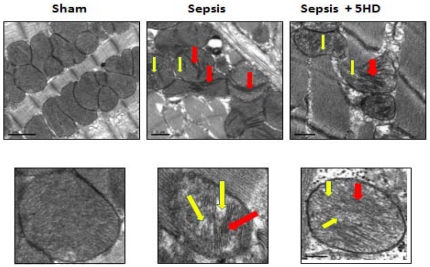
Ultrastructural changes in the mitochondrial membrane in LV tissue. **A**. Ultrastructural changes in the mitochondrial cristae (using TEM) in the left ventricular tissue section (N = 5 in each treatment group) obtained from sham and septic rat hearts(6 and 12 hr post-sepsis). The mitochondrial cristae deformation was seen in the purified mitochondrial preparation from the septic rat left ventricular tissue (6 hr post-sepsis). B. The lower panel represents the magnified image of selected mitochondria in the purified mitochondrial preparation (red box, upper panel). Arrows points to the mitochondrial cristae deformation (RED) with balloon expansion (YELLOW). Magnification, 40 k×; Scale bar = 0.1 µm.

The ultrastructural morphological features in the LV tissues obtained from the sham and septic hearts were observed using a 100 kV transmission electron microscope (Fig. 8). The image representing upper and lower panels display mitochondrial arrangements at 40× and 100× magnifications, respectively. The sham mitochondria exhibited well-defined double membranes with normal cristae arrangements. In contrast, the mitochondria from septic LV tissues had severe morphological deformations at 6 hr post-sepsis especially in the cristae with balloon-like expansions, which may be indicative of pore formation (Fig. 8). However, mitochondria from the 5HD-treated septic LV tissues exhibited morphological improvements in the cristae arrangements (Fig. 8).

## Discussion

In the present study, we demonstrated that a putative mitochondrial ATP channel blocker, 5HD, decreased sepsis-induced hypothermia, hypotension and mortality when administered at the time of sepsis induction. The results provide evidence that Bax activation is an upstream event that may precede the opening of the mitoK_ATP_, channels in sepsis. We demonstrated that 5HD reversed the sepsis-induced increased mitochondrial permeability, release of cytochrome C and ARVM contractility. These data suggest a protective effect of 5HD in sepsis-induced myocardial morbidity and mortality.

For the last several years, our laboratory demonstrated sepsis-induced myocardial contractile dysfunction in both *in vivo* and *in vitro* paradigms of polymicrobial sepsis [8], [11], [12], [22]–[27]. Sepsis-induced myocardial contractile dysfunction was found to be associated with the induction of intrinsic apoptosis cascade [8], [10], [11], [22], [23]. In the present study, we observed that the induction of sepsis in the rat was associated with a decline in body temperature (hypothermia) and mean arterial pressure with 70% mortality. The treatment of septic animals with 5HD at the time of sepsis induction not only reversed sepsis-induced hypothermia and hypotension but also decreased the mortality (20%) of septic animals. Further, reversal of sepsis-induced hypotension and decrease in mortality was seen when 5HD was administered using an Alzet pump in hypothermic (less than 89°F) septic animals. The echocardiographic analyses revealed that 5HD could partially reverse sepsis-induced decreased cardiac output, ejections fraction and fractional shortening but did not affect systemic inflammatory response (TNF-alpha levels). These data demonstrate that a putative mitoK_ATP_ blocker (when administered at the time of induction of sepsis and at 6 hr post sepsis induction) can potentially delay sepsis-induced cardiodynamic alterations and mortality in the septic rat model. Similar to the *in vivo* data, 5HD reversed sepsis-induced ARVM contractile dysfunction. As reported earlier [22], the present study also found that NE produced a blunted contractile response in the ARVMs, which was reversed by 5HD in the septic ARVMs. We observed that the percent peak shortening in both septic and sham ARVMs following NE+5HD treatment was similar in both sham and septic animals, implying protective effect of 5HD on ARVMs against sepsis-induced depressed contractile dysfunction. These results and those in our earlier reports, together, indicate that deficient contractility of NE in septic ARVMs could be due to the induction of a mitochondrial-related intrinsic apoptosis cascade.

Electron microscopy analyses of both myocardial tissues and purified mitochondrial preparations show strong evidence of cristae deformation on OMM in the septic ARVMs. We observed that OMM derangements in the septic myocardium include the presence of balloon-like expansions indicative of pore formation. Most interestingly, the 5HD-treated septic LV tissues exhibited significant gains with respect to the recovery of mitochondrial membrane and cristae morphology. These results correlate well with the loss of the mitochondrial membrane ΔΨm in the septic ARVMs, an effect reversed by 5HD. Hence, the data suggest that 5HD effectively reversed a sepsis-induced loss of mitochondrial membrane ΔΨm and OMM damage.

Our earlier studies demonstrated that, in addition to elevated caspase-3 and Bax, the intrinsic apoptosis marker cytochrome C was elevated in the cytosolic fraction of heart tissue fractions during late sepsis [8]. Similar increases in the active caspase-3 and leakage of cytochrome C in septic ARVMs have also been observed [9], [22]. Elevated Bax protein translocation to the OMM has been found to be associated with mitochondrial damage and induction of apoptosis [28], [29], [30]. In the current study, we also observed that elevated cytosolic Bax correlated with mitochondrial membrane ΔΨm collapse in the septic ARVMs. Although the sepsis-induced deterioration of mitochondrial membrane ΔΨm was reversed by the 5HD treated ARVMs, the Bax protein was still elevated in the septic ARVMs. We speculate that this increase could be due to the cytosolic regulation of Bax activation, an upstream regulator for OMM damage, while 5HD primarily provides protection against OMM damage and blocks mitoK_ATP_ channel opening. In addition, we also observed that 5HD treatment increased sepsis-induced depressed VDAC-1, suggesting that 5HD blocked sepsis-induced downregulation of mitochondrial VDAC 1. OMM VDAC1 protein is an integral part of permeability transition pore (PTP) and is a candidate for the regulation of mitochondrial permeability alterations. We observed a close association between the loss of VDAC1 and the loss of mitochondrial membrane ΔΨm in the septic ARVMs, implying that 5HD provided protection against sepsis-induced OMM damage. However, an uncompensated opening of the mitoK_ATP_ channels caused significant depolarization and rupture of the OMM, as well as loss of cytochrome C [31], [32].

The data from the present study support our speculation that during sepsis, early activation of Bax caused mitochondrial membrane ruptures that correlated with increased cytochrome C release from the outer mitochondrial membrane. It appears that Bax-mediated OMM damage could be responsible for opening the mitoK_ATP_ channels located in the inner mitochondrial membrane (IMM) leading to an uncompensated increase of cytochrome c release. We speculate that 5HD treatment acts as a patch on the damaged outer mitochondrial membrane and thus may block the release of cytochrome C and produce a stabilizing effect on OMM.

PTP in OMM have been identified as VDAC that form multicomponent complexes with adenine nucleotide translocase (ANT) and cyclophilin D located in the inner mitochondrial membrane (IMM) [33], [34]. VDACs are present in its most abundant isoform, VDAC1 in the OMM [35], [36]. Under physiological conditions, VDAC1 exists in a low-conductance state, allowing the exchange of cytochrome C and ATP between the mitochondria and cytosol [37], [38]. On the other hand, the mitoK_ATP_ channels, a distinct type of channel, are primarily located in the IMM [39], [40]. MitoK_ATP_ channels have been found to consist of Kir6.1 or Kir6.2, which are associated with sulfonylurea protein SUR 2A; this protein has a regulatory role in the sensitivity of these channels to pharmacological agents [19], [41]. Co-immunoprecipitation image analyses revealed a brilliant yellow fluorescence due to the co-localization of ANT and VDAC1 in the sham ARVMs. However, the PMPs obtained from septic ARVMs exhibited dispersed VDAC 1 (red fluorescence) and ANT (green fluorescence), suggesting that sepsis hinders the formation of VDAC 1 and the ANT complex in the mitochondria. Analyses of immunoblot data revealed that sepsis produced decreased levels of ANT and its complex with both SUR2 and VDAC1. Therefore, we speculate that sepsis may either trigger the loss of ANT, which could be responsible for a decrease in the PTP activity of controlling the release of cytochrome C in the OMM. However, further studies are needed to explore these mechanisms in detail.

In summary, this study provides evidence for the first time of the protective nature of 5HD due to the stabilizing effect on the OMM and the decreased release of cytochrome C on myocardial morbidity and mortality in a polymicrobial septic rat model. The data presented in the current manuscript, where 5HD (when administered at the time of induction of sepsis) reversed sepsis induced mortality suggest that 5HD infusion has potential to be part of infusion therapy in septic patients. In addition, 5HD can also has therapeutic implications to increase the survival in sepsis and related pathologies. However, more clinical studies may be required to explore this stipulation. We concluded that mitoK_ATP_ channel inhibitors (such as 5HD) can be a novel class of agents with the potential to delay sepsis-induced morbidity and mortality.

## Materials and Methods

### Drugs and Interventions

5-hydroxydecanoic acid (5HD, putative mitoK_ATP_ channel antagonist) and Diazoxide (putative mitoK_ATP_ agonist) were purchased from Sigma Aldrich, St. Louis, MO. 5HD (100 µmoles•kg^−1^•d^−1^/100 µL saline) was used in an Alzet pump *in vivo*, while 5HD (100 µmoles) was used for *in vitro* studies. NE (10 µmoles, GrensiaSicor Pharmaceuticals, CA) was used in the *in vitro* studies [22], [23].

### Preparation of animals

Male Sprague-Dawley rats (Harlan, IN, USA) weighing 300–350 g were used in the study. The rats were acclimatized to the laboratory conditions for at least 7 days following their arrival. All experiments were conducted in compliance with the humane animal care standards outlined in the *NIH Guide for the Care and Use of Experimental Animals* and were approved by the Institutional Animal Care and Use Committee of Baylor College of Dentistry, Texas A&M Health Science Center (Standard Operating Procedure 02-15-2007 and ARU 06-20).

### General surgical preparation and induction of polymicrobial sepsis in the rat

The rats were randomized into septic and non-septic groups at the time of surgery. Each rat was anesthetized using pentobarbital sodium (Abbott; 50 mg/kg, i.p.). Sepsis was induced in the animals using an i.p. injection of cecal inoculum as described previously [8], [9], [22], [26]. Briefly, a 0.25 cm vertical midline abdominal incision was made and rats in the sepsis group received an i.p. injection of cecal inoculum (400 mg cecal material/kg/5 mL of sterile dextrose water, D_5_W). The cecal inoculum was prepared by mixing cecal contents obtained from donor rats (euthanized with i.p. pentobarbital; 100 mg/kg) with 5% D_5_W to yield a concentration of 400 mg cecal material in 5 ml. Fresh inoculum was prepared each day, and the material from one donor rat was used within two hr for 3–5 experimental animals. The sham-septic rats received sterile D_5_W (5 ml/kg, i.p.) only. All incisions were closed with interrupted silk sutures, and the abdomen gently massaged to distribute the injectate.

### In vivo schedule for 5HD treatment

To determine the effect of 5HD on sepsis-induced hypothermia and hypotension, two separate groups based on the time schedule were tested in the present study. For the first group of sham and septic animals (n = 10 in each group), the Alzet pump containing 5HD (100 µmoles in 100 µL saline) or 100 µL saline was placed intradermally in the peritoneal skin of the sham and septic animals at the time of induction of sepsis/sham. For the second group of sham and septic animals, the 5HD (100 µmoles in 100 µL saline) was administered at 6 h post-sepsis induction, to simulate post-SIRS clinical sepsis. In both groups, the survival rate, rectal temperature and mean arterial pressure (MAP) were measured at 6, 12, 18, 24, 48 and 72 hr post Alzet pump placement.

Echocardiography was also performed on the first group of animals (N = 6 in each group); however, cardiodynamics was assessed at 6 and 12 hr post-treatment to avoid effect of anesthesia in these animals.

### Measurement of mean arterial pressure and rectal temperature

The rectal temperature was recorded at each time point using a rectal probe before measuring MAP (using the noninvasive tail cuff method;CODA, Kent Scientific) at each time point.

### Echocardiography

Under controlled anesthesia (40 mg/kg ketamine+10 mg/kg xylazine cocktail), using phased array, two-dimensional color imaging (*Vivid I*, GE Healthcare; with a 12 MHz probe or GE vivid I with 10 mHz probe) with EKG monitoring, the transthoracic echocardiographic parameters were recorded, which simulate the American Society of Echocardiography guidelines [42].

Briefly, in the first of animals (N = 6) measurements were made online with optimal digital images selected from at least 10 cardiac cycles. The left atrial and aortic diameters were obtained in the parasternal long-axis orientation, whereas the thickness of the interventricular septum, the posterior wall and left ventricular (LV) dimensions were determined in the parasternal long-short axis at the tips of the papillary muscle. Transmitral Doppler flows (E and A velocities and their ratio were measured in the apical 4-chamber or apical long axis views with the sample volume placed at the tips of the mitral leaflet. Pulmonary vein inflow was measured using pulse-Doppler in parasternal long-axis orientation after color flow localization. The LV end-systolic and end-diastolic areas were traced in single plane apical 4-chamber view. The apical 4-chamber view was used for recording tissue Doppler signals from the lateral mitral valve annulus. Standard formulas were used for echocardiographic calculations. Since an established regression formula relating LV mass to heart weights for rats remains to be established, the following formula for humans was applied because it is in agreement with published necropsy rat heart weights: LV mass = 1.04[(LVD+PW+VS)^3^−LVD^3^]×0.8+0.6. LVD is LV diameter at end diastole (onset of R wave), PW is posterior wall thickness, VS is ventricular septum thickness and 1.04 is the estimated specific gravity of the myocardium; the remaining constants are correction factors.

### Measurement of ARVM contractility

We used a well established model in our laboratory to determine the effect of 5HD on sepsis-induced ARVM contractile dysfunction, In addition, we determined the effect of 5HD on norepinephrine (NE)-induced exaggerated contractile dysfunction in sepsis [22], [23]. The mechanical properties of the ARVMs were assessed using a video-based edge detection system (IonOptix Corporation, Milton, MA) [9], [22], [25]. In brief, the ARVMs were placed in a Warner chamber mounted on the stage of an inverted microscope (Olympus, X-70) and superfused with a buffer containing (in mM): 131 NaCl, 4 KCl, 1 CaCl_2_, 1 MgCl_2_, 10 glucose, and 10 HEPES at pH 7.4. The ARVMs were isolated from sham and septic rat hearts (N = 5, atleast 50 ARVMs isolated from 5 rat hearts in each treatment group) harvested at 12 hr post sham/sepsis induction. The isolated single ARVMs were divided into four plates, which were subsequently treated with vehicle, NE, 5HD and NE+5HD. In the combination treatment group, the ARVMs were treated with 5HD 30 min prior to NE treatment administration. The mechanical properties of the ARVMs were examined at a stimulation frequency of 0.5 Hz for 20 msec using a pair of platinum wires placed on the opposite sides of the chamber connected to a FHC stimulator (Brunswick, NE). The polarity of the stimulatory electrodes was reversed frequently to avoid a possible build-up of electrolyte by-products. The ARVMs being studied were displayed on the computer monitor using an IonOptix MyoCam camera, which rapidly scanned the image area every 8.3 ms so that the amplitude and velocity of shortening/relengthening were recorded with good fidelity. Soft-edge Detection software (IonOptix) was used to capture the changes in cell length during shortening and relengthening in real time. The contractility parameter such as peak shortening (PS) was calculated using transient analysis software as described earlier [25].

### Immunoblot (Western blot) analysis

In brief, the ARVM extracts (N = 4 in each treatment group) in lysis buffer containing pepstatin (2 µg/µL, aprotinin (0.1 µg/µL), leupeptin (2 µg/µL), benzamidine (16 µg/µL) and bacitracin (0.5%) in Tris/glycine buffer were centrifuged. Following the technique of enzyme preparation, the samples were separated on 7.5% denaturing sodium dodecyl sulfate (SDS) polyacrylamide gels. The proteins were blotted onto polyvinylidene Fluoride (PVDF) membrane by electroblotting for one hour at 150 volts. The blots were blocked overnight at 4°C with 5% nonfat dry milk in tris saline buffer containing (0.2%) tween 20 and incubated with their selective primary antibody (polyclonal IgG reactive to rat proteins) for one hour at room temperature [9], [22], [23], [24]. The blots were then washed and incubated with an appropriate secondary antibody for one hour at room temperature. The specific proteins were detected by using chemiluminescence (ECL detection reagent, Amersham Pharmacia Biotech). The expression of the VDAC 1, cytochrome C, and Bax proteins was examined and normalized to β-actin/β-tubuline/HSP 60 as needed for the validation of cytosolic and mitochondrial fractions, respectively [8].

### Purified mitochondrial preparations (PMP) from ARVMs

To determine the mitochondrial-specific effects of 5HD and diazoxide, we isolated PMP from the ARVMs. Previously, ARVMs (1×10^6^ ARVMs/treatment group) were isolated from the sham and septic rat hearts harvested at 12 hr post sham/sepsis). Briefly, 800 µl of Reagent A solution was added to the ARVM pellet and incubated for 2 min. Ten µl of Reagent B was added and incubated on ice for 5 min. Then, 800 µl of mitochondrial isolation Reagent C was added, and the tubes were inverted several times to mix the liquids. The tubes were centrifuged at 700×g for 10 min at 4°C. The pellets (debris) were discarded and the supernatant centrifuged at 3,000×g for 15 min at 4°C. The supernatant (cytosolic fraction) was removed from the mitochondrial pellet and stored for further analysis. After that, 500 µl of wash buffer was added to the mitochondrial pellet and centrifuged at 12,000×g for 5 min to wash the surface of the pellet. The purified mitochondrial preparations were stored at −80°C in dry ice for further analysis.

### Measurement of mitochondrial membrane potential (ΔΨm)

To determine the mitochondrial membrane integrity, we measured the mitochondrial membrane potential using a cationic JC-1 dye. Briefly, the sham and septic ARVM pellets (1×10^6^ ARVMs/treatment group) isolated from the sham and septic rat hearts harvested at 12 hr post sham/sepsis were prepared separately and washed two times with PBS at 37°C before incubating with JC-1 reagent (500 µL). In the healthy cells, the dye stains mitochondria bright red, indicating an intact mitochondrial membrane potential. However, in the apoptotic cells, the dye remains in the cytoplasm and emits green fluorescence, indicating mitochondrial membrane collapse. A fluorescent microscope with a dual bend pass filter designed to detect fluorescein and rhodamine was used. The mitochondrial ΔΨm in the sham and septic ARVMs was assessed with a fluorescent probe, JC-1 (Molecular Probes, USA). Briefly, the ARVMs incubated with 5 µM of JC-1 for 10 min at 37°C were washed and placed on a thermostat stage at 37°C. The fluorescent images were visualized using a Nikon Optical TE2000-S inverted fluorescence microscope with excitation at 490 nm and emission at >520 nm. The acquired signal was analyzed with image-analysis software (Simple PCI). A minimum of six fields were selected and average intensity for each region was quantified. The ratio of J-aggregate to JC-1 monomer intensity for each region was calculated. A decrease in this ratio was interpreted as the loss of *ΔΨm*, whereas an increase in the ratio was interpreted as a gain in *ΔΨm*.

### Co-Immunoprecipitation technique

To determine the interaction of ANT with VDAC 1 or SUR 2, we performed immunoprecipitation, a technique offering a rapid and simple means of separating a specific protein from whole cell lysates or culture supernatants. Co immunoprecipitation of ANT/VDAC and ANT/SUR2 was performed by the Dynabeads Protein G method according to manufacturer's protocol (Invitrogen). The procedure was performed in PMPs (N = 3 in each treatment group) obtained from 1×10^6^ ARVMs/treatment group. Briefly, the PMPs were washed with ∼10 ml of PBS in a conical tube and spun at 400×g for 10 min. Five micrograms of ANT, VDAC 1 and SUR 2 antibodies were diluted in 200 µl W & B buffer (0.1 M Na Phosphate, 0.01% Tween 20, pH 8.2) and added to two different tubes containing Dynabeads Protein G. The tubes were incubated for 10 min at 25°C and washed twice with washing buffer (PBS). After washing, the Dynabeads-Ig-complex was incubated with 10 µl of PMP (sham and/or sepsis) and incubated for 10 min at 25°C. The Dynabeads-Ig-Antigen complex was subsequently washed three times in PBS and the denatured samples (70°C) eluted using a magnet. The SDS-PAGE gel electrophoreses were performed according to the standard western blot technique.

### Immunocytochemical (ICC) technique

The VDAC 1 and ANT co-localization was analyzed in the sham and septic PMP. Briefly, the PMP (specimens) were dried on glass slides and washed with 1× PBS. The specimens were incubated with 10% normal blocking serum in PBS for 20 min to suppress the non-specific binding of IgG. After washing with 1× PBS, the specimens were incubated with the primary antibodies of VDAC 1 (goat polyclonal) and ANT (rabbit polyclonal) at a concentration of 1∶200 in PBS with 1.5% normal blocking serum for 60 min at room temperature. After repeated washing with 1× PBS, the specimens were incubated for 45 min with the donkey anti-rabbit IgG-FITC (1∶100) and donkey anti-goat IgG-555 (1∶100) in PBS with 1.5–3% normal blocking serum. The specimens were then washed five times with 1× PBS and mounted on a cover slip using Slow Fade Gold (anti-fade reagent, Invitrogen) and stored in the dark at 4°C. The specimens were analyzed using a Leica SP2 confocal microscope. The secondary antibody conjugated with FITC provided green fluorescence for ANT and red fluorescence for VDAC 1.

### Electron Microscopy

To determine the ultrastructural alteration in the LV and PMPs mitochondria, we performed electron microscopy as described below. The LV tissues (N = 5 in each treatment group) were harvested and fixed in 3% glutaraldehyde from the sham and septic (6 h and 12 h) animals. In addition, PMPs were obtained, pelleted and fixed in 2.5% glutaradehyde in PBS (pH 7.4) at 4°C. The specimens were post-fixed with 2% Osmium tetroxide in 0.1 M phosphate buffer (pH 7.4), dehydrated and embedded in epoxy resin. Thin sections (∼90 nm) were harvested and placed on 200 mesh copper grid with carbon support, then stained with 2% uranyl acetate and Reynold's lead citrate. The thin sections were examined using a JEOL 1200 EX transmission electron microscope at 60 kV at a specified nominal magnification and images were captured using a 1 k×1 k Gatan CCD camera (Gatan Inc., Pleasanton, CA).

### Statistical Analyses

The hemodynamic, biochemical and protein expression data were analyzed using a two-way ANOVA repeated or one-way ANOVA (using SPSS software). After obtaining a significant F-value, a post hoc multiple range Student-Newman-Keuls test was performed. The log-rank test (Mantel-Cox) was used to test for significant differences among survival curves using Prism 5.0 software (GraphPad Software, La Jolla, CA). A probability value of p≤0.05 was considered to be statistically significant.
